# Testosterone induces off-line perceptual learning

**DOI:** 10.1007/s00213-012-2769-y

**Published:** 2012-06-17

**Authors:** Nicholas D. Wright, Thomas Edwards, Stephen M. Fleming, Raymond J. Dolan

**Affiliations:** 1 Wellcome Trust Centre for Neuroimaging, Institute of Neurology, University College London, 12 Queen Square, London, WC1N 3BG UK; 2 Department of Government, London School of Economics, Houghton Street, London, WC2A 2AE UK; 3 Center for Neural Science, New York University, 4 Washington Place, New York, NY 10003 USA; 4 Department of Experimental Psychology, University of Oxford, South Parks Road, Oxford, OX1 3UD UK

**Keywords:** Testosterone, Learning, Visual, Timescale

## Abstract

**Rationale:**

Perceptual learning operates on distinct timescales. How different neuromodulatory systems impact on learning across these different timescales is poorly understood.

**Objectives:**

Here, we test the causal impact of a novel influence on perceptual learning, the androgen hormone testosterone, across distinct timescales.

**Methods:**

In a double-blind, placebo- controlled, cross-over study with testosterone, subjects undertook a simple contrast detection task during training sessions on two separate days.

**Results:**

On placebo, there was no learning either within training sessions or between days, except for a fast, rapidly saturating, improvement early on each testing day. However, testosterone caused “off-line” learning, with no learning seen within training sessions, but a marked performance improvement over the days between sessions. This testosterone-induced learning occurred in the absence of changes in subjective confidence or introspective accuracy.

**Conclusions:**

Our findings show that testosterone influences perceptual learning on a timescale consistent with an influence on “off-line” consolidation processes.

**Electronic supplementary material:**

The online version of this article (doi:10.1007/s00213-012-2769-y) contains supplementary material, which is available to authorized users.

## Introduction

Perceptual learning refers to experience-dependent improvement in perceptual abilities (Karni and Bertini 1997). Here, we focus on two key features of perceptual learning. Firstly, dynamic regulation of perceptual learning is critical to balance adaptation to new environments against protection from irrelevant information (Seitz and Watanabe 2005). Second, perceptual learning is also characterised by its evolution across distinct timescales (Gilbert 1994), with at least two stages: a fast within-session improvement occurring over minutes; and a slow improvement triggered by practise only evident after a latent “consolidation” period (Karni and Sagi 1993; Karni and Bertini 1997). It has been argued that fast learning may reflect the setting up of a task-specific processing routine for solving the perceptual problem (Karni and Bertini 1997) and the marshalling of attention (Seitz and Watanabe 2005), whilst slow learning reflects an ongoing, perhaps structural, modification of basic representations within the processing system (Karni and Sagi 1993). However, considering these two features of perceptual learning together, it is unclear how different putative neuromodulatory systems might relate to different timescales of perceptual learning. Here, we test a for a novel candidate neuromodulator, the androgen hormone testosterone, and ask on which timescale it might act.

Testosterone is secreted in men and women, being dynamically modulated in response to environmental contingencies (Wobber et al. 2010). It is reported to have effects on diverse cognitive domains ranging from attention (Fontani et al. 2004) to social interaction (Wright et al. 2012; Eisenegger et al. 2010). In relation to learning, testosterone administration in elderly men has been shown to improve the recall of short storeys and routes (Cherrier et al. 2001), and in women to improve both spatial memory (Postma et al. 2000) as well as accuracy at mental rotation of objects (Aleman et al. 2004). However, other than prenatal testosterone enhancing quail chicks' responses to maternal calls (Bertin et al. 2009), no role in perceptual learning has previously been reported.

A priori, it is unclear on which timescale of perceptual learning testosterone might act. For example, testosterone has been previously associated with enhanced attention (Fontani et al. 2004) that might improve learning within session; whilst a slow timescale of effect might suggest a role in consolidation that would accord with testosterone’s ability to induce cellular changes as seen in rodents (Fuxjager et al. 2011). Testosterone’s widespread actions present further difficulties in parsing its possible role in perception. For example, testosterone might exert an effect on confidence, as suggested by recent work showing that testosterone increases the weighting individuals gave to their own opinions during collaborative perceptual decision-making (Wright et al. 2012). Here, to address this possibility, we use confidence ratings to examine possible changes in both metacognitive accuracy and confidence during perceptual learning.

We examined testosterone’s causal influence on visual perceptual learning. In a double-blind placebo-controlled design, we used a simple visual contrast detection task where contrast threshold provided an objective measure of visual perceptual performance. Subjects undertook the contrast detection task on two separate days, enabling us to examine its effects on learning within and between training sessions. To identify if testosterone affected measures of metacognition, after each trial, participants made a confidence rating about their decision success. We hypothesised that testosterone would improve perceptual sensitivity and would increase subjective confidence, but we were agnostic as to whether learning might occur within and/or between sessions.

## Materials and methods

### Participants

Twenty-one female participants completed the study (mean age 23 years, range 19–30). One further participant was excluded due to use of only half of the confidence rating scale, which prevented accurate estimation of metacognitive accuracy and confidence. We confined our sample to women, in whom there is prior evidence linking behavioural effects to both endogenous (Dabbs and Hargrove 1997; Archer 2006; Sapienza et al. 2009) and exogenous testosterone (Bos et al. 2010; Eisenegger et al. 2010). All were healthy with normal or corrected to normal visual acuity and took no medication other than long-standing contraceptives (10 participants took combined oestrogen and progestogen contraception; two took progestogen-only contraception). All reported regular menstrual cycles (29 ± s.d. 2.4 days, range 28–35 days) and were tested between days 1–14 of their cycle. A second, control, experiment was conducted with 10 female participants (mean age 23, range 19–28). All gave written informed consent and the experiment was approved by the local ethics committee.

### Experimental procedure

**Fig. 1:**
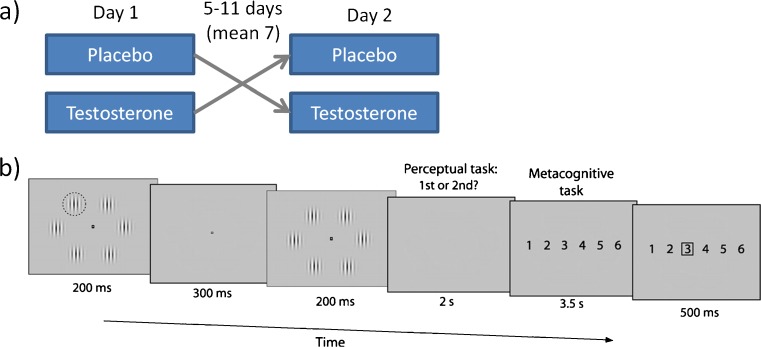
Experimental design. **a**) Participants attended on two separate days in a blinded, randomised, placebo-controlled cross-over design. **b**) Participants completed a two-alternative forced-choice task that required two judgments per trial: a perceptual response followed by an estimate of relative confidence in their decision. The perceptual response indicated whether the first or second temporal interval contained the higher-contrast (target) Gabor patch (highlighted here with *a dashed circle* that was not present in the actual display), which could appear at any one of six locations around a central fixation point. Target Gabor contrast was continually adjusted with the use of a staircase procedure to maintain ~71 % correct responses—and this provided a measure of performance as contrast threshold. Confidence ratings were made using a one-to-six scale, with participants encouraged to use the whole scale from one = low relative confidence to six = high relative confidence. The *black square* in the rightmost panel indicates the choice made in the metacognitive task

In a randomised, placebo-controlled, double-blind, cross-over design, 80 mg testosterone undecanoate was administered orally (Restandol® testocaps™; Fig. 1a). Ten participants received testosterone then placebo, and 11 received placebo then testosterone. Oral testosterone undecanoate is widely used clinically and has well known pharmacokinetics (Houwing et al. 2003). To provide a washout period, participants attended on two separate days, 5–11 days apart (mean 7 days ± 1 s.d.). Given testosterone’s circadian rhythm, all participants attended at the same time on both testing days: 08:45 a.m. and 3:00 p.m. On each testing day, at 08:45, the participant received testosterone/placebo then left the laboratory and returned at 15:00 for the behavioural task. Prior to treatment, to aid absorption, all participants had consumed or were given a moderate breakfast. The hormonal manipulation was identical to that used in a previous study, which caused an eight-fold increase in testosterone at the time of testing relative to either baseline or placebo (Wright et al. 2012).

In a control experiment, participants attended on two separate days, 3–7 days apart, (mean 5.4 days ± 1.8 s.d.) without receiving any treatment. In the control experiment, participants only attended the laboratory once on each day (for behavioural testing), and the time of attendance was not constrained to occur at a specific time of day.

### Behavioural methods

#### Task

In each trial, participants first made a visual judgement that comprised a temporal two-alternative forced-choice pop-out task (Fig. 1b). In one interval, all six Gabor gratings had the same contrast, but in the other interval, one of the Gabors was of higher contrast (the “pop-out”). The temporal interval and spatial position of the pop-out Gabor varied randomly between trials. Participants had to decide in which interval the pop-out appeared and input their choice using the left hand with the numbers “1” or “2” on the QWERTY keypad of a standard PC keyboard. Accuracy was held at approximately 71 % using a 1-up 2-down staircase procedure, in which the contrast of the pop-out Gabor was chosen from a stimulus set of pop-out Gabors (see below). Our task provides an objective measure of perceptual performance, given by the contrast threshold adjusted by the staircase. Perceptual learning would lead to a reduced contrast threshold required to maintain 71 % correct responses.

To investigate timescale effects, we examined learning within and between training sessions. Participants underwent identical procedures on both days: first, performing a short practise session to familiarise themselves with the stimuli and task; and next performing the main experiment that consisted of 600 trials, split into six blocks of 100 trials. On both days, all subjects had the same high-contrast (i.e. easy) start-point for the staircase in the first block, and then for each subsequent blocks, the contrast of the pop-out Gabor at the end of each block was used as the starting contrast for the pop-out Gabor in the next block. Due to this, imposed start-point block 1 was analysed separately from blocks 2–6. Consequently, when reference is made to behaviour within a session, this refers to blocks 2–6. No feedback was given on individuals’ performance.

Our design enabled us to isolate effects of testosterone on both objective perceptual learning and subjective confidence judgements (Fleming et al. 2010). In each trial, after making their judgements, participants indicated their confidence in a perceptual decision they had just made on a scale of 1 (low relative confidence)–6 (high relative confidence) using their right hand to press one of the numbers “1”–“6” on the numerical keypad. A square red frame (width 1°, thickness 0.1°) appeared around the selected rating. Participants were instructed to try to use the whole confidence scale and to bear in mind that the scale represents relative confidence for that day as, given the difficult nature of the task, they would rarely be completely certain that their visual judgement had been correct.

#### Stimuli

The perceptual decision display comprised six Gabor gratings (circular patches of smoothly varying light and dark bars) arranged around a central fixation point (Fig. 1). Each Gabor subtended 1.4° of visual angle in diameter, and consisted of a luminance pattern modulated at a spatial frequency of 2.2 cycles per degree. Each “baseline” Gabor had a contrast of 20 % of maximum, and appeared at a mean eccentricity of 6.9°. The fixation point comprised a black square measuring 0.2° across, luminance 0.10 cd/m^2^, with a central white square 0.1° across, luminance 13.64 cd/m^2^. The background was a uniform grey screen of luminance 3.66 cd/m^2^.

Baseline Gabors were displayed with a contrast of 20 % (where 0 % is no difference between the luminance of the grating bars and 100 % is maximum difference, i.e. black to white). The pop-out Gabors were drawn from a stimulus set in which contrast varied from 23 to 80 % in increments of 3 %. At the time of confidence ratings, the display consisted of a grey screen (luminance 3.66 cd/m^2^) with the numbers 1–6 written left to right (luminance 13.64 cd/m^2^, 0.7° in height, centred around fixation).

Stimuli were presented on a gamma-calibrated CRT display (Dell FP2001, 20.1 inch display; 800 × 600 pixels; 60 Hz refresh rate) at a viewing distance of approximately 60 cm, situated in a darkened room. Stimulus display and response collection were controlled by Matlab 7.8.0 (Mathworks Inc., Natick, MA, USA) using the COGENT 2000 toolbox (http://www.vislab.ucl.ac.uk/cogent.php).

### Data analysis

Contrast threshold was quantified as the mean contrast level maintained by the staircase in each of blocks 2–6. Awareness of performance was quantified by computing a measure of metacognitive accuracy from participants’ confidence ratings in blocks 2–6, as reported previously (Fleming et al. 2010). Specifically, we computed measures of metacognitive accuracy (*A*_roc_) and bias (*B*_roc_) from the Type 2 receiver operating characteristic.

Statistical tests were carried out using paired or independent-samples *t* tests, or mixed analyses of variance (ANOVA) in SPSS 17.0; reported *p* values are two-tailed.

## Results

**Fig. 2:**
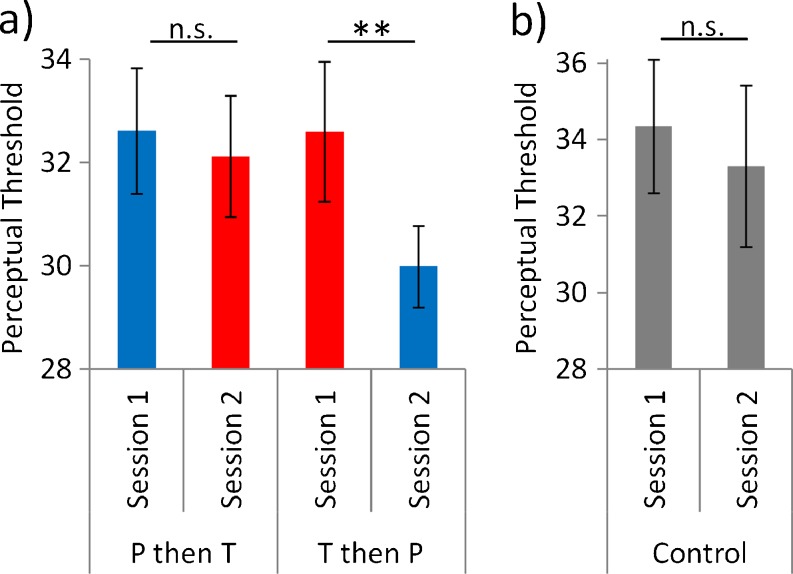
Testosterone-induced “off-line” perceptual learning between sessions. Better performance is indicated by a lower contrast threshold. **a**) Individuals who performed the task on day 1 under Testosterone (*T*) subsequently exhibited markedly improved performance when they were performing the task on day 2 when they received Placebo (*P*). No improvement was seen when individuals conducted the task under P on day 1 and then under T on day 2. **b**) One possibility here is that instead of T inducing learning in the group who received T then P, T affected the group who received P then T instead by preventing the expression of learning in the latter group on day 2. We address this possibility in a control experiment with a separate group of subjects, in whom no treatment was given on either day, with no difference observed in threshold between. *Error bars* indicate s.e.m.; *double asterisks* indicate *P* = 0.01

Our staircase achieved good control over participants’ proportion of correct responses (71.7 ± 1.0 %). Testosterone induced perceptual learning between training sessions (Fig. 2a). Individuals who received testosterone on day 1 showed a markedly reduced threshold on day 2 (T then P: day 1 threshold mean 32.6 ± 4.3 s.d.; day 2, 29.2 ± 2.5; *t*_(9)_ = 3.3, *P* = 0.01). This learning between sessions only occurred under testosterone, as no improvement was seen in the group who received placebo on day 1 and then testosterone on day 2 (P then T: day 1, 32.6 ± 4.0; day 2, 32.1 ± 3.9; *t*_(10)_ = 1.1, *P* > 0.3). The learning induced by testosterone between sessions is shown in Fig. 2a and is summarised using contrast threshold as the dependent variable in a two-treatment (Testosterone, Placebo) by two-order (T then P, P then T) mixed ANOVA (main effect of treatment *F*_(1,19)_ = 5.52, *P* = 0.030; interaction *F*_(1,19)_ = 11.88, *P* = 0.003).

One possibility is that rather than inducing learning in the group who received the active agent and then placebo, testosterone affected the group who received placebo followed by testosterone instead, by preventing the expression of learning in the latter group on day 2. We address this possibility in a control experiment with a separate group of subjects, in whom no treatment was given on either day. Data from the control experiment failed to support this alternative hypothesis, as no difference was observed in threshold between days (day 1, mean 34.4 ± 5.5 s.d.; day 2, 33.3 ± 6.7; *t*_(9)_ = 1.3, *P* = 0.23; Fig. 2b).

**Fig. 3:**
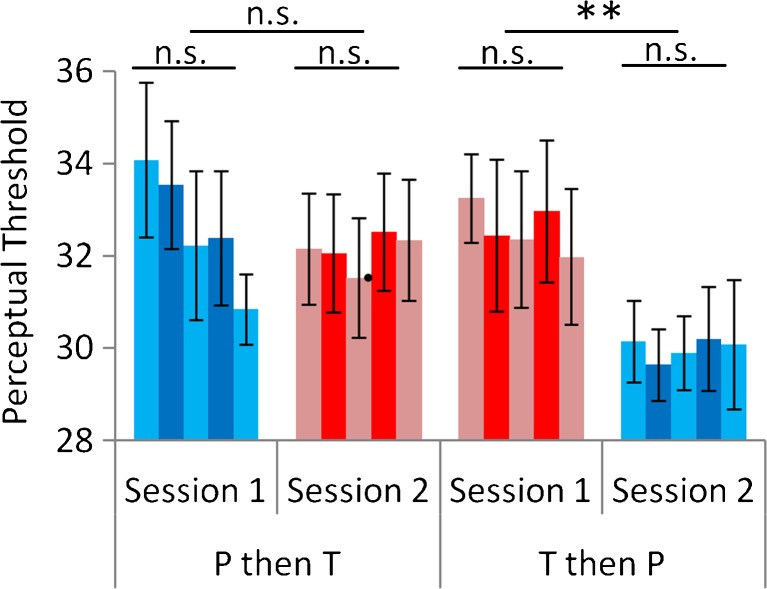
Testosterone did not induce perceptual learning within sessions. There was no change in contrast threshold within training sessions (having excluded block 1) as a function of treatment or day (one-way ANOVAs revealed no effect of block (2–6) on either day under either treatment, details in main text). Indeed, learning between sessions is still seen even when including block as an extra factor (see main text). Note that the trend towards improvement within session on day 1 under placebo was not replicated under placebo in day 2, and also cannot explain the clear between-session learning we find. *Error bars* indicate s.e.m.; *double asterisks* indicate *P* = 0.01

Given the distinct timescales on which perceptual learning occurs, we next examined learning within training sessions. There was no change in contrast threshold within training sessions (having excluded block 1) as a function of treatment or day (Fig. 3; one-way ANOVA revealed no effect of block (2–6) for day 1 under testosterone *F*_(4,36)_ = 0.85, *P* = 0.50; day 2 placebo *F*_(4,36)_ = 0.01, *P* = 0.98; day 1 placebo *F*_(4,40)_ = 2.4, *P* = 0.063; or day 2 testosterone *F*_(4,40)_ = 0.4, *P* = 0.77). Stability in performance within sessions is further illustrated using contrast threshold as the dependent variable in a two-treatment (P, T) by five-block (2 to 6) by two-order (T first or P first; between-subjects factor) mixed ANOVA, in which there was a main effect of treatment (*F*_(1,19)_ = 5.5, *P* = 0.030); an interaction of treatment by order (*F*_(1,19)_ = 11.88, *P* = 0.003); no main effect of block (*F*_(4,76)_ = 7.93, *P* = 0.14); and no interaction of block with either treatment (*P* = 0.74) or order (*P* = 0.67) or both (*P* = 0.18). We also replicated this lack of change in contrast threshold within training sessions in the control experiment (one-way ANOVA revealed no effect of block (2–6) on either day 1 *F*_(4,36)_ = 0.39, *P* = 0.82 or day 2 *F*_(4,36)_ = 1.39, *P* = 0.26). Although the start-point for contrast in block 1 was the same for all subjects, thus rendering assessment of early learning effects only tentative, for completeness, the block 1 data is illustrated in Supplementary Fig. 1.

Our task enabled us to ask whether testosterone affected measures of metacognition. Collapsing across treatments, introspective accuracy was highly stable between days within individuals (*A*_roc_ day 1 v. day 2, r = 0.53, *P* = 0.01), as was the bias in confidence judgements (*B*_roc_ r = 0.90, *P* < 1 × 10^−6^) and perceptual performance (contrast threshold r = 0.83, *P* = 4 × 10^−6^). Further, the task also successfully dissociated objective perceptual performance from both confidence-related measures (*P* ≥ 0.4 for all correlations between threshold and either *A*_roc_ or *B*_roc_ on either day 1 or day 2). Our data revealed no effect of testosterone on either *A*_roc_ (two-treatment by two-order mixed ANOVA with *A*_roc_ as dependent variable: no main effect of treatment *F*_(1,19)_ = 0.74, *P* = 0.40; no interaction *F*_(1,19)_ = 0.39, *P* = 0.54) or on *B*_roc_ (mixed ANOVA with *B*_roc_ as dependent variable, no main effect of treatment *F*_(1,19)_ = 0.006, *P* = 0.94; no interaction *F*_(1,19)_ = 0.91, *P* = 0.35). Furthermore, our findings regarding objective perceptual performance (i.e. threshold) were unaltered if either *A*_roc_ or *B*_roc_ were included as covariates in the analyses above.

Finally, we note that the results of the analyses above with respect to threshold, *A*_roc_ or *B*_roc_, were not altered when including hormonal contraception as a between-subjects factor (either with two levels [on or off contraception] or three levels [off, combined, progesterone only]). Similarly, including participants’ beliefs about which drug had been administered did not alter the results. Including time between testing sessions (days) as a covariate in the above analyses rendered the main effect of treatment on threshold non-significant, but did not alter the learning effects on threshold or the other results.

## Discussion

We show that testosterone induces an experience-based perceptual improvement. Testosterone contrasts with the ascending neuromodulatory systems previously associated with perceptual learning (Seitz and Watanabe 2005), differing markedly in its synthesis, regulation and mechanisms of action that include intracellular binding-induced transcription (Janowsky 2006). The latter mechanism is likely to be important given out observation that testosterone impacts on learning between rather than within training sessions, a timescale that strongly indicates an effect on consolidation (Robertson et al. 2004). In addition, consolidation can take two forms, an enhancement of skills and a stabilisation of memories, and here, testosterone would appear to act via the former in what is often called “off-line” learning (Robertson et al. 2004). Such slower learning is thought to depend on structural modification of basic representations within the processing system (Karni and Sagi 1993) rather than mechanisms linked to faster learning like the setting up of task-specific processing routines (Karni and Bertini 1997) or attentional mechanisms (Seitz and Watanabe 2005). More generally, delineating which neuromodulators act on which timescales may help dissociate the neural processes supporting perceptual learning.

At a cellular level, consolidation involves transcription-dependent synaptic plasticity (Dudai 1996), and this accords with the known mechanism of action of testosterone (Janowsky 2006). This may well be a generic learning effect as for example in studies of rodent learning, a surge of testosterone induces transcription and cellular changes that influence future behaviours (Fuxjager et al. 2010, 2011). In regions such as hippocampus, androgen-deprived rats and monkeys show markedly decreased synaptic density, which can be normalised with testosterone replacement (Leranth et al. 2003, 2004). Further, animal data shows that testosterone may at least in part exert its effects through local aromatisation to oestrogen (Trainor et al. 2006), which itself has important effects on neuroplasticity (McEwen 2010). Testosterone may also exert negative feedback effects on hypothalamic–pituitary hormones, such as luteinising hormone recently implicated in rodent spatial memory (McConnell et al. 2012), with such negative feedback effects potentially occurring on a timescale consistent with consolidation processes.

In humans, testosterone is associated with memory function. In healthy elderly men, testosterone supplementation improves recall of short storeys and routes (Cherrier et al. 2001) and improves working memory (Janowsky et al. 2000). In healthy young women, it induces improved spatial memory (Postma et al. 2000) and accuracy at mental rotation of objects (Aleman et al. 2004). One possibility is that testosterone may gate plasticity during training, as has been proposed for the ascending neuromodulators dopamine and acetyl-choline (Roelfsema et al. 2010). We note that with our experimental manipulation of testosterone that levels are likely to have returned to baseline before participants slept on the night of testing, with an elimination half-life of approximately 2 h (Houwing et al. 2003), suggesting that gating of plasticity may explain its effects on learning in our experiment. However, effects of exogenous testosterone are also reported around 4 h after blood serum maxima are reached (Tuiten et al. 2000; Bos et al. 2010), suggesting future work could usefully explore a potential role for sleep in testosterone-related consolidation.

Much previous work on the regulation of perceptual learning has focussed on the ascending neuromodulatory systems, which can respond very rapidly to environmental contingencies (Seitz and Dinse 2007). For example, in animals pairing dopamine release with sounds has been shown to remodel representations in primary auditory cortex (Bao et al. 2001). Furthermore, it has been proposed that these ascending neuromodulatory systems may enhance perceptual learning by enhancing attention (Seitz and Watanabe 2005; Seitz and Dinse 2007; Roelfsema et al. 2010; Rokem and Silver 2010). However, whilst endogenous testosterone has been associated with attention (Fontani et al. 2004), this seems unlikely to be the mediating factor in the visual perceptual learning we show here, as testosterone caused an improvement between sessions but did not affect performance within sessions.

Our data also address the possibility that testosterone affects confidence, as suggested by recent work showing that testosterone increased the weighting individuals gave to their own opinions during collaborative perceptual decision making (Wright et al. 2012). Such effects of testosterone on confidence might also explain why city traders make higher profits on days when they have higher morning testosterone (Coates and Herbert 2008): increased overall confidence may lead to more risk-taking; or increased accuracy in ascribing confidence to judgments (i.e. metacognitive ability; Fleming et al. 2010) may lead to superior decision making. However, we show no effect of testosterone on either overall *B*_roc_ or *A*_roc_.

However, whilst our data do not support a role for testosterone in confidence, two extensions of our design could further test this potential relationship. Firstly, we did not provide trial-by-trial feedback as this constituted a further task aspect on which testosterone might act. We believe that the presence or absence of feedback would be an interesting added factor to explore in future work. Second, in line with recent work (Fleming et al. 2010), we used a staircase procedure to hold the proportion of correct responses stable, and thus examine testosterone’s possible metacognitive effects in isolation to its effects on proportion correct. To the extent that metacognitive confidence is a function of performance, this may explain minimal effects of confidence in our design. By contrast, when performance is allowed to vary, neuromodulators may have joint effects on both performance and confidence, as recently found for dopaminergic stimulation (Lou et al. 2011).

Finally, it is important to note three limitations of our design that can be usefully addressed in future work. Firstly, our cross-over design is not optimal to dissociate effects of testosterone in the first and second sessions. In addition to the two conditions in our cross-over design (T then P; and P then T), to be fully balanced future work could add two further conditions (P then P as in our control experiment but perfectly matched; and T then T). Secondly, we note the relatively small sample size in our control experiment. Third, we only examined visual contrast detection, whilst future work could examine more general effects of testosterone on other aspects of visual learning (e.g. motion), other perceptual domains (e.g. auditory) and motor learning.

Our data identify testosterone as exerting an important influence on perceptual learning. The physiological characteristics of this neuromodulator differ markedly from those of neuromodulators previously shown to act on perceptual learning (Seitz and Watanabe 2005). Further, our data suggest that testosterone acts through “off-line” consolidation processes, which has again not been previously shown. More generally, our data hint that distinct neuromodulators may impact on learning at different timescales, providing a fruitful means to dissociate component processes supporting perceptual learning.

## Electronic supplementary material

Below is the link to the electronic supplementary material.

ESM 1 (DOCX 56 kb)
