# Supplementary material for: Testosterone induces off-line perceptual learning
Source: Psychopharmacology (Berl). 2012 Jun 17;224(3):451–7. doi: 10.1007/s00213-012-2769-y (PMC3496538; doi:10.1007/s00213-012-2769-y)
Supplement: Supplementary file 1 — (DOCX 56 kb) [file 213_2012_2769_MOESM1_ESM.docx]

## Supplementary materials:

## Testosterone induces off-line perceptual learning

Nicholas D Wright, Thomas Edwards, Stephen M Fleming and Raymond J Dolan


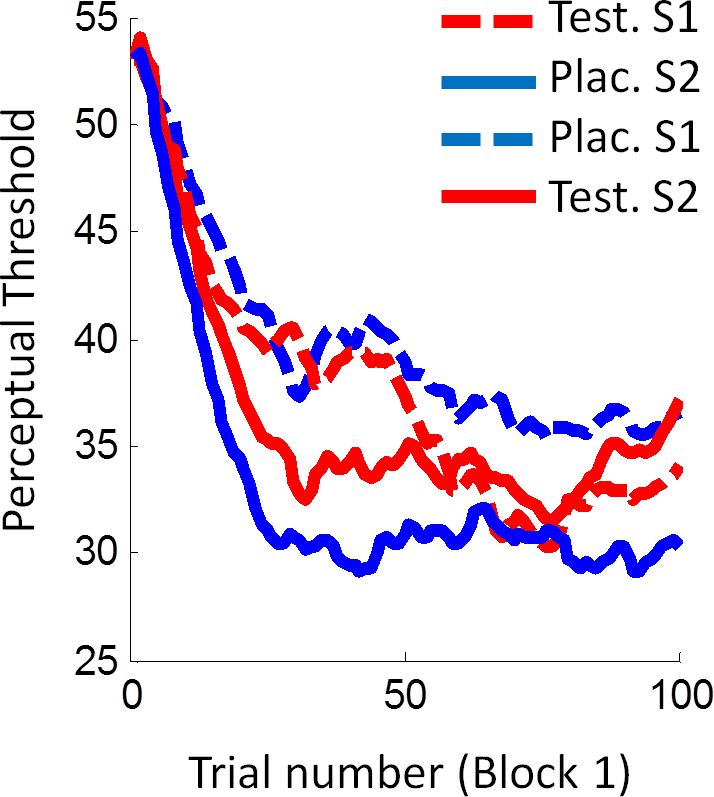


***Supplementary Figure 1 Data from Block 1.*** Better performance is indicated by a lower contrast value. This plot shows the data from Block 1, in which all subjects started at the same initial contrast level on the first trial and this was reduced by the staircase procedure over the block (for illustration a 3 trial running average is shown). The perceptual learning induced by testosterone was evident early in the first block. This is shown by the superiority of the solid blue line by early in the first block, which represents the performance on Day 2 of participants who had received T on Day 1.
